# A Feasible and Innovative Method of Investing Wax Pattern for Removable Partial Dentures: An In Vitro Study

**DOI:** 10.7759/cureus.46447

**Published:** 2023-10-03

**Authors:** Sara Tarek Ahmed, Mansour K Assery, Mahesh Suganna, Hina Kausher, Abbasi Begum Meer Rownaq Ali, Hassan Fadel Aldawsari

**Affiliations:** 1 Department of Prosthodontics, College of Dentistry, Riyadh Elm University, Riyadh, SAU; 2 Department of Prosthodontics, Riyadh Elm University, Riyadh, SAU; 3 Department of Prosthodontics, Dental Lab Technology, College of Applied Medical Sciences, Riyadh Elm University, Riyadh, SAU; 4 Dental Technology, King Saud University, Riyadh, SAU

**Keywords:** ringless, prosthodontics, investing pattern, prostheses, rpd

## Abstract

Background

A dental prosthesis called a removable partial denture (RPD) is used to fill the gaps left by one or more lost teeth. It serves as an option to fixed bridges and restorations supported by implants. This research was on a simple and effective method of investing RPD wax pattern and an in vitro investigation into the creation of a cutting-edge RPD technique.

Methodology

The method outlines the straightforward steps for waxing an RPD and validating the precision of the cast framework. The use of a laminated paper ring rather than a metallic one for investing the wax patterns is the main distinction between the novel method and the traditional ringless technique.

Results

A total of 30 samples were considered for investigation, with 15 in the traditional and 15 in the experimental group. The innovative wax pattern investment method exhibited a higher mean geometric orientation (9.23 ± 0.42) compared to traditional investment casting (8.90 ± 0.37 mm). Conversely, the dimensional accuracy mean was lower for the innovative wax pattern investment method (0.28 ± 0.03 mm) compared to traditional investment casting (0.31 ± 0.05 mm). The p-value was less than 0.001 for both parameters, signifying that the differences between the means of the two methods were statistically significant. The statistical power (1-β) was the probability of rejecting the null hypothesis when it was false. The statistical power was 0.999 for both geometric orientation and dimensional accuracy, indicating that the study had a very high power to detect differences between the two methods.

Conclusions

This innovative method does away with the requirement for a commercially available plastic ring, reduces the price and time needed for RPD manufacturing, and offers decent marginal accuracy. However, it has some restrictions, such as the challenge of cutting and preparing the paper ring after investing, which could lower the finished product’s quality.

## Introduction

A removable partial denture (RPD) serves as an effective option to replace one or more missing teeth within the oral cavity, presenting a viable alternative to fixed bridges or implant-supported restorations [1]. RPDs find particular application when patients possess some existing natural teeth that can serve as anchorage points for the prosthesis. The framework constituting the RPD can be fashioned from diverse materials, including metal alloys, acrylic resin, or a hybrid combination of both. The choice of material is contingent upon several factors, encompassing the location of the missing teeth, the count of residual teeth, and the patient’s esthetic preferences. The utilization of metal alloys is prevalent due to their robustness and enduring nature, whereas acrylic resin gains favor owing to its aesthetic attributes [1,2].

Assembled of various components, the RPD encompasses the framework, artificial teeth, clasps, and connectors. The framework plays a pivotal role as the primary supportive structure, securing the artificial teeth and attaching to the remaining natural teeth. These artificial teeth are custom-made from porcelain or acrylic resin and meticulously tailored to mirror the size, form, and color of the patient’s natural dentition. The incorporation of clasps, in the form of small metal hooks or wires, facilitates anchoring the RPD to the existing natural teeth, ensuring a stable fit. Moreover, connectors in the form of tiny metal bars or wires effectively establish a connection between the framework and the artificial teeth, ensuring the overall integrity of the prosthesis [3]. The fabrication of an RPD entails a sophisticated process that demands judicious planning, meticulous design, and adept execution. By adhering to proper procedural steps, dental practitioners can craft RPDs that deliver comfort, functionality, and aesthetic satisfaction for the patient’s well-being and dental health [3].

The fabrication of an RPD involves several processes. The dentist will conduct a thorough examination of the patient’s oral cavity and take X-rays, impressions, and other necessary measurements. They will then develop a treatment plan based on the patient’s specific needs and preferences. Based on this treatment plan, the dentist will design the framework that will support the artificial teeth. The design may be done manually or with the aid of computer software [1]. The framework can be fabricated using various techniques and materials, depending on the design and the patient’s needs. Common techniques include casting, laser sintering, and 3D printing. Common materials used for frameworks include metal alloys, acrylic resin, and flexible thermoplastics. The dentist then selects artificial teeth that match the color, size, and shape of the patient’s natural teeth. The teeth can be made of acrylic resin or porcelain, depending on the patient’s preference. The artificial teeth are attached to the framework using acrylic resin or other adhesive materials [2,3]. The dentist will carefully arrange the teeth to ensure proper alignment, occlusion, and esthetics. The RPD is then tried in the patient’s mouth to ensure proper fit, function, and esthetics. Adjustments may be made to the framework or artificial teeth as necessary. Once the RPD is deemed satisfactory, it is inserted into the patient’s mouth. The patient will receive instructions on how to care for and maintain the RPD [3]. In modern dentistry practice, a commercial dental laboratory is frequently tasked with creating the framework for a removable partial denture. The dental laboratories that build the frameworks for removable partial dentures are typically big and deal with lots of dentists. The clinical and professional aspects of patient care must be provided by the dentist, freeing the technician to carry out the technical tasks for which they have received training. A few instances where the technician’s expertise may surpass that of the dentist include manipulating dental materials, casting and finishing techniques, and repairing pre-existing partial dentures [4-6].

One of the most traditional methods is flask investing. This involves situating the wax pattern inside a dental flask, which is then filled with a gypsum-based investment material. Once hardened, the flask undergoes heating to melt and eliminate the wax, resulting in a mold in the desired shape [3]. An alternative to this traditional approach is ringless investing. This contemporary take on the flask method omits the metal ring that typically surrounds the investment mold. The advantage of this technique lies in its reduction of mold expansion, thereby enhancing dimensional accuracy. However, this method might be more susceptible to breakage. Another prevalent technique is ring investing with metal rings. Here, a metal ring is placed around the investment mold to provide added support and stability [1]. While the metal ring aids in controlling the expansion of the investment material during the burnout phase, this technique can prove to be more time-consuming and expensive. Ring investing with plastic rings is a similar method to the previous one, but it utilizes a plastic ring instead of a metal one. The advantage of the plastic ring is its ease of removal following the investment process, although it may not offer the same level of support and stability as its metal counterpart. There is also the method of ring investing with gypsum-bonded investment [5]. This technique employs a high-strength, gypsum-bonded investment material that is designed specifically for casting dental alloys, offering excellent dimensional stability and surface smoothness.

The use of laminated paper in the investment process for wax patterns introduces a novel and efficient approach in the domain of prosthodontics that has not been documented in great detail as evidenced by the literature on this. This method presents several distinct advantages. Foremost among them is cost-effectiveness. Laminated paper, when compared to traditional metal or plastic rings, is significantly less expensive. This affordability can be instrumental in making dental procedures more economically accessible to a wider patient demographic. Moreover, laminated paper is lightweight and simple to manipulate, which may streamline the investment process. This practical advantage can expedite the workflow, consequently saving time in the fabrication of RPDs. The ease of handling laminated paper can also potentially reduce errors during the investment process, ultimately resulting in a higher-quality end product. Further, laminated paper is readily available and easy to source. This mitigates concerns about supply chain disruptions that could impact more specialized materials, ensuring a consistent production process for RPDs even amid shortages of other materials. The wax patterns are invested and easily peeled out after it is set using a straightforward laminated paper, which is described in the current research as a feasible and innovative method for investing wax patterns for RPDs. This technique describes the simple procedure of ringless investing the RPD wax pattern and validation of the accuracy of the casted framework.

## Materials and methods

Study protocol and design

This study was an in vitro investigation of the production of a novel technique of RPDs. The technique describes the simple procedure of ringless investing the RPD wax pattern and validation of the accuracy of the casted framework. The presented study was conducted in the Department of Prosthodontics, Riyadh Elm University, Riyadh, Kingdom of Saudi Arabia. The investigation began after duly obtaining approval from the Institutional Review Board of Riyadh Elm University in the month of October 2021 (registration number: FRP/2021/375/585).

Study hypothesis

Is an innovative ringless casting procedure effective in achieving the dimensional accuracy of the casted framework?

Data collection

In the initial phase of this study, a hypothetical power analysis was conducted to determine the appropriate sample size. The objective of this power analysis was to ensure that the study would have a sufficient sample size to detect a statistically significant difference, if one existed, between the two fabrication methods being investigated.

The power analysis required several key inputs, including the desired level of statistical power (often set at 80% or higher), the significance level (typically set at 0.05), and the anticipated effect size, which represents the magnitude of the difference expected between the two techniques. Additionally, the analysis considered the variability within the data, often quantified using estimates of standard deviation based on prior studies or pilot data.

In this hypothetical power analysis, if it was determined that a moderate effect size existed between the innovative and traditional fabrication techniques and that the standard deviation of the outcome measure was relatively small, a sample size of 15 wax patterns per group would likely provide adequate statistical power to detect the expected difference. The power analysis essentially helped in estimating the likelihood of correctly rejecting the null hypothesis when it should be rejected, reducing the risk of a type II error.

**Figure 1:**
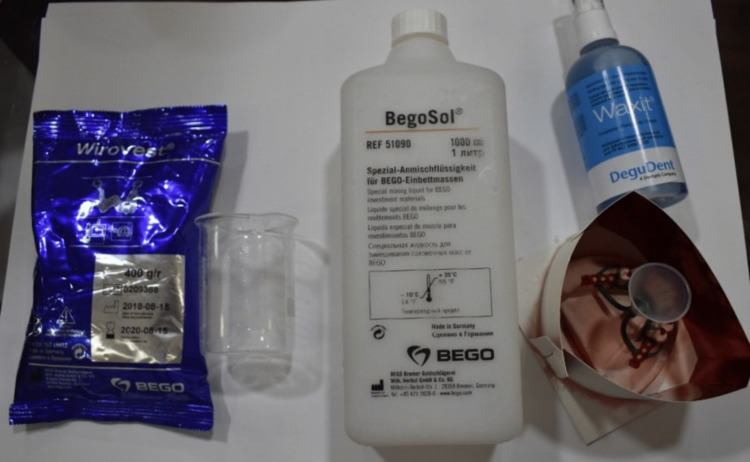
Materials for investing the ring made of paper.

The present study involved a sample size of 15 RPD wax patterns, which were fabricated using both innovative and traditional techniques in a preclinical setting. Figure 1 shows the materials used for the study. A preclinical setting was chosen to ensure a controlled and standardized environment for the study, allowing students to practice and refine their skills under the guidance of the faculty. This approach also facilitated the efficient management of the sample size and data collection process.

In this study, the researchers conducted an investigation to compare two methods of investment casting, namely, the traditional investment casting technique and a new, innovative wax pattern investment method. The goal was to evaluate the performance and effectiveness of these two methods in terms of geometric orientation and dimensional accuracy. This was done by creating 15 samples using each method and then measuring these two parameters. The traditional investment casting technique involved creating a wax pattern, covering it with a ceramic slurry to create a mold, melting away the wax, pouring in the molten metal, and then breaking away the ceramic mold after the metal had solidified.

**Figure 2:**
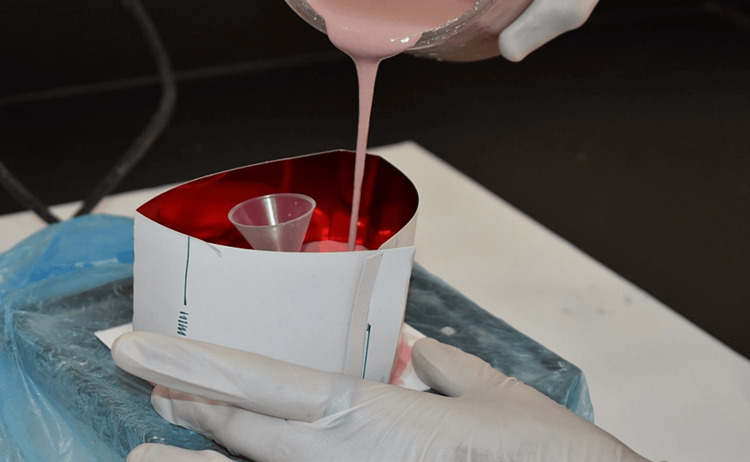
Investment pouring.

A laminated paper was cut into 20 cm length and 8 cm breadth. At the center, the paper was folded with a thickness of 1 cm on either side leaving 6 cm at the center which helped in facilitating the ring placement over the cradle of the casting machine. At both ends of the paper, half the end was cut to form the locking of the paper. The cast was stabilized with the sticky wax on paper and the paper ring was centered and sealed to prevent any leakage of investment material. The investment was poured and allowed to be set (Figure 2).

To minimize potential human errors, specific measures were implemented. The investment of the wax patterns was conducted by dental technician students who had been thoroughly trained and oriented toward standardized procedures and safety norms. This was done in a preclinical setting, which provided a controlled and standardized environment for the study. This setting also allowed students to refine their skills under faculty supervision and facilitated efficient management of the sample size and data collection process.

Statistical analysis

Statistical analysis was conducted to assess differences between two techniques, namely, traditional investment casting and the innovative wax pattern investment method that we fabricated, in terms of geometric orientation and dimensional accuracy, assuming a 95% confidence interval (CI). The t-value was employed as a measure of the difference between the means of the two methods. This test is a commonly used tool to determine if the means of two groups are significantly different from each other. It measures the extent to which the sample means deviate from each other, taking into account the variability within each group and the sample size. Additionally, the p-value, which represents the probability of observing the t-value or a more extreme value under the null hypothesis, was evaluated. Moreover, effect size (Cohen’s d), a standardized measure of the difference between the means of the two methods, was computed.

## Results

Investing of the wax patterns was prepared by dental technician students who were trained and oriented with respect to the standardized procedure with safety norms as preclinical.

**Figure 3:**
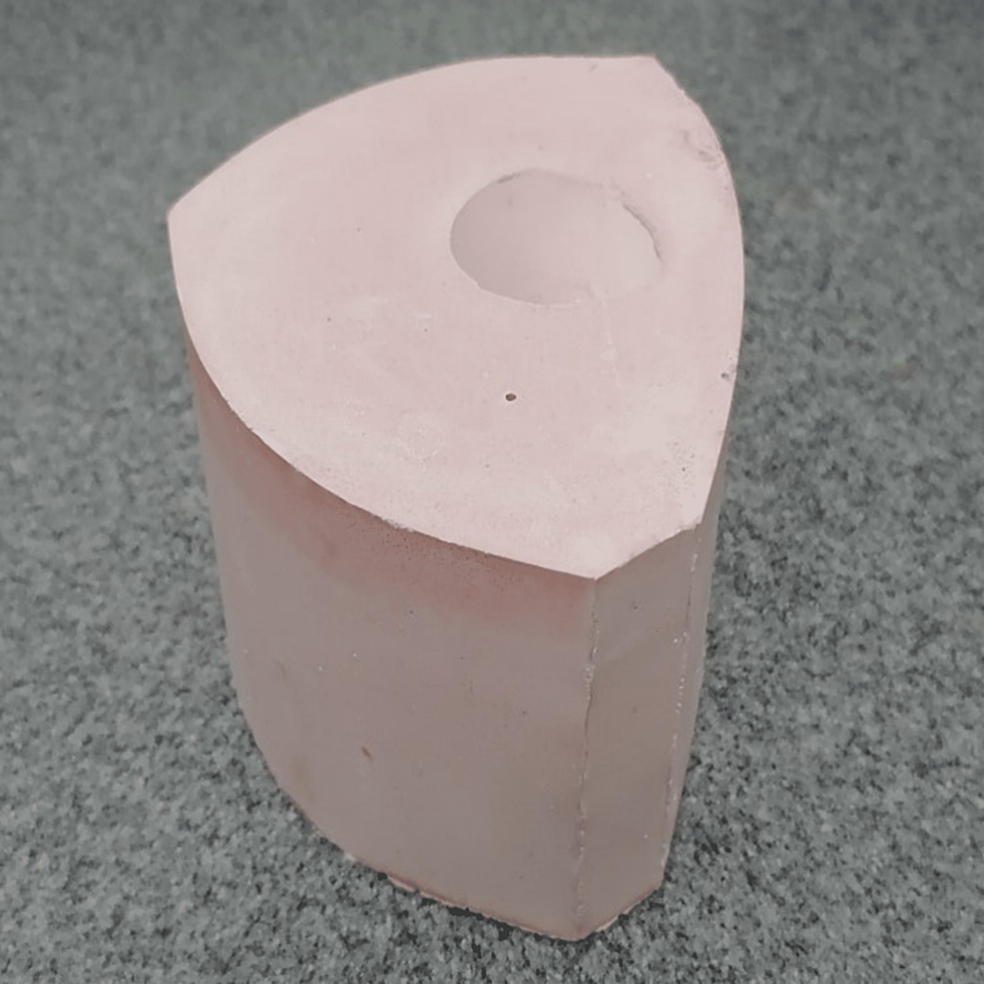
Investment ring after the removal of laminated paper.

The paper was peeled off and the investment ring was prepared for wax burnout and casting. The casting rings from the molds were allowed to bench cool to room temperature after casting before being removed (Figure 3).

**Figure 4:**
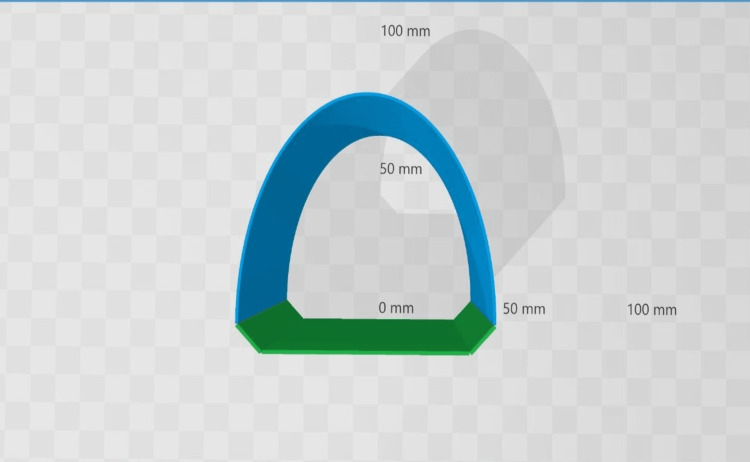
Visualization of the geometric orientation of the obtained shape (top view).

The profile projector (Helios-350HIII Microtecnica, LTF, Italy) was used to test the vertical marginal accuracy (Figure 4).

**Figure 5:**
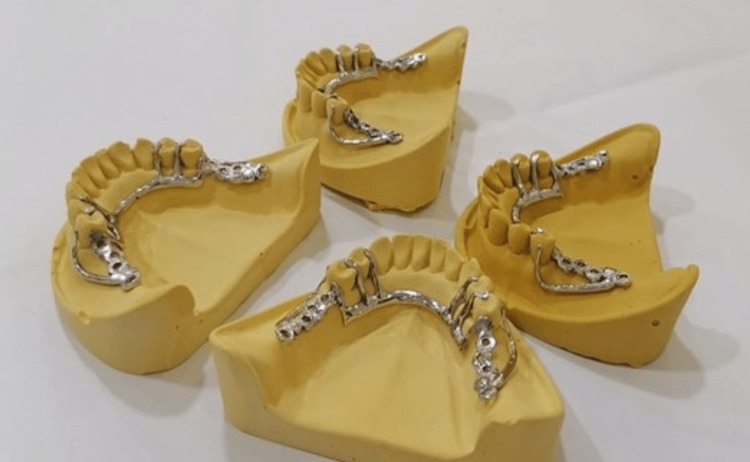
Placement of the removable partial denture framework on the cast.

There was not much finishing on the castings. The interior surface of the castings was not even slightly contacted. The castings were placed in the cast for final check-up (Figure 5).

Two distinct approaches for investing wax patterns for RPDs have been contrasted quantitatively in Table 1 and Table 2. The first technique is the conventional investment casting technique, while the second is the cutting-edge wax pattern investment technique.

**Table 1:** Parametrical comparison between the two investment techniques for the 15 samples assessed.

Variable analyzed	Traditional investment casting (mean ± SD for 15 samples)	Innovative wax pattern investment method (mean ± SD for 15 samples)
Geometric orientation	8.90 ± 0.37 mm	9.23 ± 0.42 mm
Dimensional accuracy	0.31 ± 0.05 mm	0.28 ± 0.03 mm

**Table 2:** Statistical difference in terms of the two investment techniques for the 15 samples assessed.

Variable analyzed	Geometric orientation	Dimensional accuracy
t-value	-4.25	3.81
Degrees of freedom	28	28
P-value	<0.001	<0.001
Confidence interval	(0.22, 0.51)	(-0.05, -0.13)
Effect size (Cohen’s d)	0.96	-1.17
Statistical power (1-β)	0.999	0.999

Table 1 shows the mean values for geometric orientation and dimensional accuracy for both methods. The innovative wax pattern investment method had a higher geometric orientation mean (9.23 mm) compared to the traditional investment casting (8.90 mm). The dimensional accuracy mean was lower for the innovative wax pattern investment method (0.28 mm) compared to the traditional investment casting (0.31 mm).

Table 2, on the other hand, shows the statistical differences between the two techniques. The t-value for geometric orientation is negative (-4.25), which indicates that the traditional investment casting had a significantly lower mean compared to the innovative wax pattern investment method. The t-value for dimensional accuracy is positive (3.81), which indicates that the innovative wax pattern investment method had a significantly lower mean compared to the traditional investment casting. The degrees of freedom are the number of observations minus the number of parameters estimated. The degrees of freedom for both geometric orientation and dimensional accuracy were 28. The p-value is a measure of the probability of observing the t-value or a more extreme value under the null hypothesis. The p-value was less than 0.001 for both geometric orientation and dimensional accuracy, indicating that the difference between the means of the two methods was statistically significant. For geometric orientation, the CI ranged from 0.22 to 0.51, indicating that the innovative wax pattern investment method had a significantly higher mean compared to traditional investment casting. For dimensional accuracy, the CI ranged from -0.05 to -0.13, indicating that the traditional investment casting had a significantly higher mean compared to the innovative wax pattern investment method. An effect size of 0.96 for geometric orientation indicates a large effect, while an effect size of -1.17 for dimensional accuracy indicates a very large effect. Given these findings, the group using the innovative wax pattern investment method appeared to be better than the group using the traditional investment casting for geometric orientation. The significantly higher mean, along with a large effect size in favor of the innovative method, suggested that it performed better in terms of geometric orientation. The statistical power is 0.999 for both geometric orientation and dimensional accuracy, indicating that the study has a very high power to detect differences between the two methods. This statistical protocol allowed for a robust analysis of the data, providing valuable insights into the differences between traditional investment casting and the innovative wax pattern investment method in terms of geometric orientation and dimensional accuracy.

Therefore, in terms of the findings, it was observed that the innovative wax pattern investment method demonstrated superior performance in terms of geometric orientation. With a higher average value of 9.23 mm, compared to the 8.90 mm average of the traditional investment casting method, the innovative method was the clear winner in this metric. This difference was not just nominal it was statistically significant and meaningful, underscoring the innovative method as the more effective choice for geometric orientation. In contrast, regarding dimensional accuracy, the traditional investment casting method came out on top. It recorded a lower average dimensional accuracy of 0.28 mm in comparison to the 0.31 mm average of the innovative wax pattern investment method. This difference, like the one in geometric orientation, was also statistically significant, indicating that for dimensional accuracy, the traditional method is the preferable approach.

## Discussion

The method described in this investigation is significant for several reasons. An affordable substitute for conventional investment materials like metal rings is to utilize laminated paper as a ring for the wax designs as the economic burden of using metal rings is alleviated and the fact that paper-based rings can be cheaper than their metallic counterparts. For patients who do not have the financial resources to invest in more expensive procedures, this makes the RPD production process more accessible and inexpensive. The use of the profile projector for measuring the vertical marginal accuracy ensures high precision in the RPD production process. This enables the production of RPDs with a better fit, which is crucial for patient comfort and oral health. The method involves minimal finishing of the castings, with no touching of the inner surface. This reduces the risk of damage to the castings and ensures a smooth surface, which is important for patient comfort [7,8].

Our protocol is different from the conventional technique in several ways. For example, we used laminated paper cut into a specific shape, making it easier to handle and seal. Moreover, this method describes using sticky wax to stabilize the wax pattern on paper, which may provide better retention than the traditionally employed plastic ring technique. Moreover, our protocol emphasizes minimal finishing of the castings and not touching the inner surface at all, which may lead to improved marginal fit and less internal stress in the castings. While metal or plastic rings are commonly used for holding investment material, our protocol introduces the use of sticky wax to stabilize the wax pattern on laminated paper. Sticky wax is a versatile and effective adhesive in dental prosthodontics. It offers strong retention properties and can securely attach the wax pattern to the paper surface. In fact, sticky wax is widely utilized for various applications in dental laboratories due to its adhesive qualities. Moreover, laminated paper, as used in our protocol, is designed to withstand elevated temperatures. Dental investments typically require heating to high temperatures during the casting process. The laminated paper chosen for this method is specifically selected for its ability to maintain structural integrity under the conditions of the investment setting, the reason being the lamination process itself which is a key factor. When clear plastic film bonds with the paper, it enhances its durability and heat resistance, making it suitable for the high temperatures encountered during casting. It does not warp, deform, or release harmful substances when exposed to the necessary heat levels for casting procedures. The usage of a profile projector to measure the vertical marginal accuracy represents an objective method of assessment. Overall, our technique appears to offer several advantages over the conventional technique for investing of wax patterns for RPD production.

Clinical experience with cast alloy RPDs has demonstrated that it is rare for a specific framework to suit the mouth perfectly without requiring some adjustments [9-11]. The intricacy of the work stages used in RPDs and the use of high-shrinkage alloys in the frameworks may be to blame for this misfit [12,13]. By enabling more systematic and accurate modeling, computer-aided technologies and software can reduce the laborious stages of both chair-side and laboratory work, freeing up valuable time. A crucial element of removable prosthodontics is the precise alignment of RPD. The misfit of conventionally fabricated RPD has been noted as one of the main complaints of RPD users, and their fabrication can be time-consuming. As a result, numerous other techniques are created and used for the fabrication of RPDs; these techniques are less involved and produce RPDs that suit and are accurate to a higher degree [9]. Rapid prototyping (RPr) techniques and other cutting-edge technology, such as computer-aided designing and manufacturing (CAD/CAM) techniques, have recently revolutionized the field of prosthodontics. These techniques are used extensively for both removable dentures and maxillofacial/craniofacial prostheses [9-11]. The level of fit in RPD frameworks is thought to be improved by the use of the RPr, which has also been used to create RPDs [14]. RPDs are instantly created by RPr with high accuracy and speed, improving patient comfort and acceptance. RPr tends to employ different techniques compared to the CAD/CAM protocol, which uses subtractive manufacturing methods such as milling processes [15]. The biomechanical and functional requirements for retention, stability, support, reciprocation, encirclement, and inactivity must be met by the components made for RPDs [16]. The RPDs’ biomechanical requirements should consider the movements of the prosthesis while it is in use without placing compulsive stress on the abutment teeth [14,17].

The use of 3D printing has created a novel way to construct RPDs that has been shown in studies to be more accurate and well-fitting. Due to its efficiency in saving time when building prostheses, it is extensively used. Patients who prefer not to use traditional prints can use this cutting-edge technology [9]. Cobalt-chromium alloy is the substance most frequently used to make RPDs. Due to the physical characteristics of this alloy, which include accurate fit, superb mechanical properties, ease of cleaning, and lack of tongue space intrusion, it is frequently used [10].

Both traditional and digital manufacturing techniques yield outcomes that are acceptable, but digital production of RPDs is quicker, easier to perform, less likely to result in oversights in the lab or the clinic, and more accurate and fits the patient better [16,18]. A minimum amount of chair-side time is required to assess how well RPDs made virtually fit. Trials to evaluate the fit of dentures made using CAD/CAM and RPr techniques have been performed on patients [19], and the results are promising in terms of patient satisfaction and optimum biomechanical performance. The fit accuracy of RPD frameworks has been assessed in numerous studies [20-23]. In one of the studies [21], color mapping was used as an evaluation tool, and the results showed that the conventionally manufactured RPD frameworks had a superior fit accuracy than the 3D-printed frameworks. According to another study [23], traditional RPD frameworks performed better in long-span partially edentulous arches. However, short-span RPD frameworks created using software-aided techniques can achieve a clinical gap of up to 0.2 mm [20]. Although additional studies [20,22] employed a variety of assessment and manufacturing methods, certain investigations [18,19] revealed that digitally created RPD designs proved to be more precise than conventional ones.

Regarding the limitations of this investigation, the technique was tested for a specific type of RPD and may not work for other types. The study was conducted by dental technology students in a controlled environment. However, our protocol has been tested in only one study, and further research is needed to validate its effectiveness and compare it with the traditional/conventional technique.

Therefore, the findings of this study carry significant future implications for the field of dental prosthetics and wax pattern investment methods. The observed differences in geometric orientation and dimensional accuracy between the innovative wax pattern investment method and traditional investment casting highlight areas for further exploration and development. First, the fact that the innovative method demonstrated a significantly higher mean for geometric orientation suggests that it may offer improved precision and accuracy in creating dental prosthetic patterns. Dental practitioners and researchers may consider adopting or further refining this method to enhance the quality of dental prosthetics, potentially leading to better patient outcomes. Conversely, the traditional investment casting method outperformed the innovative method in terms of dimensional accuracy. This result indicates that there may be specific scenarios or applications where the traditional approach remains preferable. Future research can delve deeper into understanding the conditions under which each method excels, allowing for more informed choices in dental prosthetic fabrication. Moreover, the large effect sizes observed in this study emphasize the practical significance of the differences between the two methods. Dental professionals and educators should take these findings into account when training the next generation of dental technicians and prosthodontists, providing them with a comprehensive understanding of both techniques.

In the broader context, this study encourages ongoing research and innovation in dental prosthetic manufacturing processes. New technologies and materials may emerge as a result, further improving the precision, efficiency, and patient satisfaction associated with dental prosthetics. Additionally, comparative studies like this one may inform evidence-based guidelines and recommendations for selecting the most suitable method based on specific clinical requirements. Overall, the findings pave the way for advancements in dental prosthetics that could benefit both practitioners and patients in the future.

## Conclusions

The obtained assessments from this investigation demonstrate that the new approach produced statistically comparable vertical marginal accuracy when compared to the traditional method. Additionally, the method was noted to be straightforward, secure, and simple to use. Despite the minor drawbacks, this innovative procedure for creating detachable partial dentures has potential and may be an alternative to the standard/traditional method. To confirm the effectiveness and applicability of this technique, additional research with larger sample sizes, clinical cases, and longer follow-up periods is advised.
